# Brilliant Cresyl Blue Negative Oocytes Show a Reduced Competence for Embryo Development after In Vitro Fertilisation with Sperm Exposed to Oxidative Stress

**DOI:** 10.3390/ani13162621

**Published:** 2023-08-14

**Authors:** Lilli Bittner-Schwerda, Carolina Herrera, Sarah Wyck, Eleni Malama, Christine Wrenzycki, Heinrich Bollwein

**Affiliations:** 1 Clinic of Reproductive Medicine, Vetsuisse Faculty, University of Zurich, 8057 Zuerich, Switzerland; 2 Veterinary Clinic for Reproductive Medicine and Neonatology, Chair for Molecular Reproductive Medicine, Justus-Liebig-University Giessen, 35392 Giessen, Germany

**Keywords:** oocyte quality, DNA damage, sperm quality

## Abstract

**Simple Summary:**

Oxidatively stressed sperm can fertilise oocytes and, hence, transfer its damaged genome to a progeny. Sperm cannot repair its own genome, therefore the oocyte’s capacity to deal with damaged sperm influences the outcome of a fertilisation. We distinguished bovine oocytes of different qualities in vitro using Brilliant Cresyl Blue (BCB) staining and in vitro fertilised them with non-oxidised and oxidised bovine semen. BCB-negative oocytes were less able to support embryo development after fertilisation with oxidatively stressed bovine semen, demonstrated by lower cleavage and blastocyst rates, a delay in development and lower blastocyst quality. These results support a further description of the oocytes’ quality in regard of their capacity to deal with damaged sperm, which will help in selecting the best oocytes when damaged sperm have to be used in assisted reproduction.

**Abstract:**

The extent of oxidative damage transferred by the damaged sperm to the progeny is likely to be limited by the oocyte’s repair and antioxidative capacity. We aimed to assess the association between Brilliant Cresyl Blue (BCB) staining in oocytes and their competence for embryo development after in vitro fertilisation (IVF) with damaged sperm. For this purpose, bovine sperm were incubated without (non-oxidised sperm, NOX S) or with 100 µM H_2_O_2_ (oxidised sperm, OX S) and were used to fertilise in-vitro-matured bovine oocytes (BCB-pos./BCB-neg.). Unstained oocytes served as controls (US). Development was assessed at 30, 46, 60 h and on Days (D) 7 and 8 after IVF. Total cell number and apoptotic index were analysed in D7 blastocysts. BCB-neg. oocytes showed lower cleavage rates and blastocyst rates than unstained oocytes after IVF with NOX S (*p* < 0.05). They showed the highest reduction in D7 blastocyst rate upon fertilisation with OX S and showed a delayed embryo development at 46 and 60 h after IVF compared to embryos produced with NOX S (*p* < 0.05). Total cell number in blastocysts produced with BCB-neg. oocytes was lower (*p* < 0.05) in the embryos produced with OX S than in embryos after IVF with NOX S. In conclusion, BCB-neg. oocytes have a lower competence to support embryo development after in vitro fertilisation with oxidised sperm.

## 1. Introduction

Sperm cells are especially vulnerable to oxidative stress because of their limited anti-oxidative capacities and large amounts of polyunsaturated fatty acids in their plasma membrane [1,2]. An excess of reactive oxygen species (ROS) damages a variety of pivotal molecules such as lipids [3], polysaccharides [4], proteins [5] and DNA [6]. There is no doubt that high levels of ROS in sperm induce plasma membrane alterations [7] and hamper motility [8]. At lower levels of ROS, however, sperm might retain their ability to fertilise the oocyte [9,10], which means a transfer of damaged paternal molecules or toxic metabolites to the oocyte. The extent of oxidative damage and toxic metabolites transferred by the sperm to the progeny is likely to be limited by the oocyte’s repair and antioxidative capacity [11]. Mature sperm have limited antioxidative capacities and cannot repair damaged DNA, as they only possess truncated DNA damage repair mechanisms [12]. In consequence, insults in the paternal genome must be repaired after fertilisation to ensure the transfer of intact genetic material to the progeny [13]. Furthermore, toxic molecules introduced must be detoxified. Antioxidative defence and DNA repair in the newly fertilised oocyte relies entirely on mRNAs and proteins stored in the oocyte [14,15,16,17]. Just after the major embryonic genome activation, at around the eight- to sixteen-cell stage in cattle, the embryo itself contributes to DNA damage repair and regulation of the redox state [18,19]. If the oocyte is not adequately equipped, or if embryonic genome activation fails or is delayed, the embryo will arrest [20]. Which factors define an oocyte’s capacity to deal with oxidatively damaged sperm has not been finally determined so far. The donor’s age, oocyte’s size and environmental factors are suggested to play a role, as large oocytes in cattle [21], in-season oocytes of trout [11] and oocytes of young mice [22] were able to support embryo development after fertilisation with irradiated or oxidatively damaged sperm better than their counterparts. Also, other markers of oocyte quality as, for example, spindle imaging, anti-Stokes Raman scattering microscopy and cumulus cell transcriptomics and proteomics have been described [23]. Brilliant Cresyl Blue (BCB) staining indicates the activity of the Glucose-6-phospate dehydrogenase (G6PDH), an enzyme which is part of the pentose phosphate pathway and provides ribose phosphate for nucleotide synthesis and NADPH for the reduction of, for example, glutathion (GSH), which is an important antioxidant [24]. Considering the importance of G6PDH in supplying the oocyte with antioxidative factors, we aim to assess if there is an association between BCB staining of the oocytes and their competence for embryo development after fertilisation with oxidatively damaged sperm. At the same, time negative effects of the used quality marker itself have to be avoided. A further description of oocyte quality in regard to its repair capacity of damaged sperm would possibly aid a better selection of valuable oocytes for assisted reproductive techniques and is further deepening the understanding of the repair capacity to pave the way for future studies; for example, aiming on supporting the oocyte’s repair capacity [25].

## 2. Materials and Methods

### 2.1. Chemicals

Unless otherwise stated, all chemicals were obtained from Sigma-Aldrich (Buchs, Switzerland).

### 2.2. In vitro Production of Bovine Embryos

#### 2.2.1. Oocyte Collection

**Figure 1:**
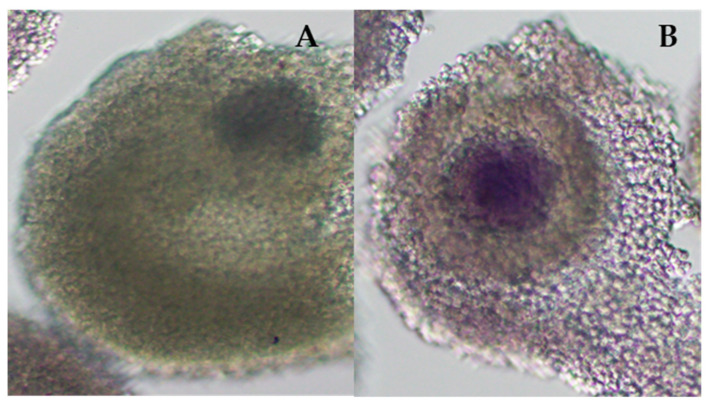
Bovine COCs after staining with Brilliant Cresyl Blue (BCB). (**A**) BCB-negative (neg.) oocytes were colourless. (**B**) BCB-positive (pos.) oocytes coloured blue.

Bovine ovaries from slaughtered animals were bought at the local abattoir, kept at 38 °C in 0.9% NaCl and transported to the lab within 2 h. The slicing method was used to retrieve Cumulus–oocyte complexes (COCs) [26] and were collected into modified PBS (mPBS) (containing 0.036 mg/mL pyruvate, 0.05 mg/mL streptomycin, 1 mg/mL glucose, 0.13 mg/mL CaCl_2_, 0.06 mg/mL penicillin G and 0.5 mg/mL heparin sulphate). Two morphological categories of COCs were selected for in vitro culture: COCs with a homogenous, evenly granulated cytoplasm possessing at least three layers of compact cumulus cells and COCs with less than three layers of cumulus cells, or partially denuded COCs but with a homogenous evenly granulated cytoplasm. All other morphological types of COCs were discarded. Selected COCs were pooled in holding medium, which consisted of Hepes-buffered TCM-199 supplemented with 10% (*v*/*v*) foetal bovine serum, and were subsequently split into two groups. One group was stored in mPBS with 0.4% BSA at 38 °C in humidified air as unstained oocytes. The other group was incubated in mPBS containing 0.4% BSA and Brilliant Cresyl Blue (0.01 mg/mL) for 90 min at 38 °C in humidified air. Following incubation, COCs were separated based on blue nuclear staining. The BCB-positive (pos.) oocytes, coloured blue, and BCB-negative (neg.) oocytes (Figure 1), which were colourless, as well as unstained oocytes, were washed in in vitro maturation media (IVMm), which was TCM-199 supplemented with 2.1 mg/mL Na-bicarbonate, 10% (*v*/*v*) foetal bovine serum, 0.2 mM sodium pyruvate, 1 mM glutamine, and 1 mg/mL pFSH. They were then allocated to groups of 10 for in vitro maturation.

#### 2.2.2. In Vitro Maturation

COCs were matured in vitro in 50-µL droplets of IVMm (in groups of 10), under mineral oil for 20 to 24 h in 5% CO_2_ in air and saturated humidity, at 38.2 °C. For all following steps, oocytes of different groups (BCB-pos., BCB-neg., unstained oocytes) were handled separately, but at the same time.

#### 2.2.3. In Vitro Fertilisation

Following in vitro maturation, the expanded COCs were rinsed in Hepes-TALP (Hepes-buffered TALP medium supplemented with 6 mg/mL BSA) and placed into 100 µL microdroplets of Fert-TALP (TALP medium supplemented with 6 mg/mL BSA, 20 mM penicillamine, 10 mM hypotaurine and 0.2 mg/mL heparin sulphate) under mineral oil (in groups of 20).

The cryopreserved semen used for fertilisation was from a bull with proven fertility (blastocyst rate day (D) 8 after IVF: 27.5%). Semen straws contained 250 µL of semen with 60 million sperm per mL and were thawed for 30 s in a 37 °C water bath. Subsequently, sperm were washed in a 90% Percoll^®^ Plus column. For that, semen samples were deposited on top of a 750 µL Percoll solution and centrifuged for 15 min at 600× *g*, at room temperature (RT). The pellet was removed and re-suspended in 200 µL Hepes-TALP. For induction of oxidative stress in sperm, 100 µM H_2_O_2_ was added to the oxidized sperm (OX S) sample. Both, non-oxidised sperm (NOX S) and OX S were incubated for 1 hr at 37 °C. The effect of incubation and treatment with 100 µM H_2_O_2_ on semen motility and DNA fragmentation for the bull used in these experiments has been published before [27]. Following the incubation, sperm were washed twice for 3 min at 600 g with Hepes-TALP and then re-suspended in 100 µL IVF medium. Sperm cells were added to a final concentration of 2 × 10^6^ sperm/mL to the IVF microdroplets containing different classes of oocyte qualities (unstained, BCB-pos., BCB-neg.). All groups (NOX S and OX S) were inseminated at the same time. The day of IVF was defined as day 0 (D0) of culture. Incubations were carried out for 18 to 24 h in 5% CO_2_ in air and saturated humidity, at 38.2 °C.

#### 2.2.4. In Vitro Culture

Presumptive zygotes were rinsed and cultured for 24 h in 50 µL microdroplets of SOF0m (synthetic oviduct fluid [28] supplemented with 1 mM glutamine, 2% (*v*/*v*) BME-essential amino acids, 1% (*v*/*v*) MEM-non essential amino acids, and 8 mg/mL BSA–FAF [29]) in groups of 10, in 5% CO_2_, 5% O_2_ at 38.2 °C in saturated humidity. In the next step, embryos were transferred into SOF1.5m (same as SOF0m + 1.5 mM glucose) and stripped of cumulus cells. The number of inseminated oocytes undergoing cleavage was recorded. Further culture was performed in 50 µL microdroplets of SOF1.5m at 38.2 °C, 5% CO_2_ and 5% O_2_ under mineral oil (in groups of 10). Cleavage rate and stage of development were registered at 30, 46 and 60 h (Percentages of embryos at different cell stages as 2-, 4-, 6/8-, >8-cell stage) after IVF. On D7 and D8, the blastocyst rate was evaluated. In total, 2278 oocytes were cultured (per oocyte/sperm combination group in mean 280 oocytes (Min 235, Max 587). To minimise handling of oocytes, cleavage rates at 30 h after IVF and stages of development at 46 and 60 h after IVF were determined in a limited number of in vitro production (IVP) cycles, using 553 oocytes for the evaluation of the cleavage rates at 30 h after IVF and 1005 oocytes for assessment of stages of development at 46 and 60 h after IVF. The percentage of early-cleaving embryos (≤30 h after IVF) was calculated as percentage of embryos cleaved at 30 h relative to the number of embryos finally cleaved at 46 h after IVF. In order to investigate specific effects mediated by paternal oxidative stress exposure, changes in developmental rates upon fertilisation with OX S was calculated per OC quality group as follows: 100-(developmental rate [cleavage rate, blastocyst rate] with OX S/developmental rate [cleavage rate, blastocyst rate] with NOX S).

### 2.3. Evaluating Embryo Quality Using the TUNEL Assay

Number of total cells per embryo and cells presenting signs of nuclear DNA fragmentation in blastocysts was evaluated using the TUNEL (Terminal deoxynucleotidyl transferase dUTP nick end labelling) assay. The TUNEL assay was performed as described previously by Byrne and Southgate (1999) using an in situ cell death detection kit (Roche Diagnostics, Mannheim, Germany) [30]. Blastocysts on D7 were fixed using 4% paraformaldehyde and washed in PBS. Then, embryos were permeabilised by 0.1% Triton X-100 in PBS for 30 min at RT followed by a transfer to the TUNEL reaction mixture for 1 hr at 38 °C, after which they were washed in PBS. Nuclei were stained using Hoechst (2 mg/mL 3342 Bisbenzimid). Embryos were washed in PBS and mounted on glass slides using Vectashield Antifade mounting media (Vector Laboratories, Burlingame, CA, USA). Embryos for the positive controls were incubated before in 50 U/mL DNase I (Roche, Basel, Switzerland) for 1 hr at 38 °C. Negative controls were incubated in the absence of the terminal deoxynucleotidyl transferase. Embryos were finally covered with coverslips. Coverslips were pressed on the slides in order to even the blastomeres and allow a better separation and counting of cell nuclei. Labelling of TUNEL and Hoechst nuclei was observed using an inverted LEICA CTR6000 microscope with a Leica Microsystems LAS-AF6000 (Leica Microsystems, Bensheim, Germany). Each embryo was analysed for the total cell number (blue nuclei) and TUNEL-positive blastomeres (green) with DAPI and FITC filters, using a 200× magnification. Analysis was performed blind to the oocyte group and sperm treatment group. Only embryos in which single blastomeres were well-defined and where minimal overlay of nuclei was present were included in the analysis. The apoptotic index was calculated as percentage of green nuclei relative to the total number of nuclei per embryo. The TUNEL assay was performed in 60 embryos of 5 independent IVP cycles.

### 2.4. Statistical Analysis

All Data were analysed using Graph Pad Prism 4.0 (Graph Pad Software Inc., San Diego, CA, USA). Normal distribution was tested using the Shapiro–Wilk normality test. Data in which at least one group was not normally distributed were analysed using non-parametric tests. In the case of analysing the effects of the sperm group (NOX S vs. OX S) within the same oocyte class, the Mann–Whitney test was applied. When effects (developmental rates, decrease in developmental rate, timing of embryo development and embryo quality) between oocyte quality classes (unstained oocytes, BCB-pos. oocytes, and BCB-neg. oocytes) were assessed and data were not normally distributed, the Kruskal–Wallis test followed by the Dunn’s multiple comparison test were carried out. Normally distributed data were compared using the one-paired *t*-test, in the case of analysing the effects of sperm treatment, and the one-way analysis of variance (ANOVA) followed by the Tukey’s multiple comparisons test, in the case of analysing the effects of oocyte quality class. A *p*-value of < 0.05 was considered as significant.

## 3. Results

### 3.1. Embryo Development and Timing of Development after Fertilisation with NOX S

**Figure 2:**
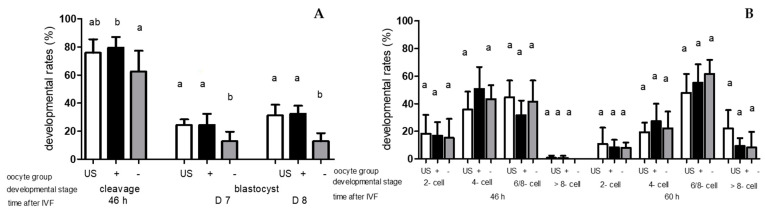
(**A**) Cleavage rates (46 h after IVF) and blastocyst rates on Days (D) 7 and 8 after IVF and (**B**) Percentages of embryos at different cell stages (2-, 4-, 6/8-, >8-cell stage) at 46 and 60 h after IVF of unstained oocytes (US) and BCB-pos. oocytes “oocyte group +” and BCB-neg. oocytes “oocyte group –“, fertilised in vitro with non-oxidised sperm (NOX S). Values are means + SD. Different letters “a, b” indicate differences (*p* < 0.05) between oocyte groups fertilised with NOX S.

The percentages of BCB-pos. oocytes and BCB-neg. oocytes were 45.75% ± 6.90% and 54.25% ± 6.90%, respectively (*p* ≥ 0.05). After IVF with NOX S, the BCB-neg. oocytes showed significantly lower cleavage rates than BCB-pos. oocytes (*p* < 0.05), while no differences (*p* ≥ 0.05) were noticed between unstained oocytes and BCB-pos. oocytes, and between unstained oocytes and BCB-neg. oocytes. Fertilisation with NOX S caused lower blastocyst rates on D7 and D8 (*p* < 0.05) in BCB-neg. oocytes compared to unstained oocytes and BCB-pos. oocytes. Blastocyst rates on D7 and D8 from BCB-pos. oocytes and unstained oocytes were similar (*p* ≥ 0.05) after IVF with NOX S (Figure 2A). The early cleavage rate (≤30 h after IVF) was higher (*p* < 0.05) in embryos produced with BCB-stained oocytes and NOX S compared to embryos produced with unstained oocytes and NOX S (Table 1). Furthermore, BCB-pos. oocytes showed more embryos (*p* < 0.05) cleaving early than BCB-neg. oocytes after fertilisation with NOX S. There was no difference (*p* ≥ 0.05) in embryo developmental stages at 46 h and 60 h after IVF between embryos from different oocyte classes produced with NOX S (Figure 2B).

### 3.2. Embryo Development and Timing of Development after Fertilisation with OX S

**Figure 3:**
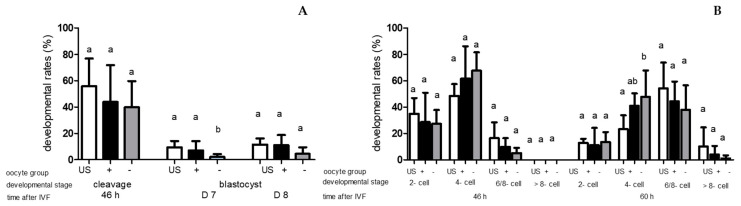
(**A**) Cleavage rates (46 h after IVF) and blastocyst rates on Days (D) 7 and 8 after IVF and (**B**) Percentages of embryos at different cell stages (2-, 4-, 6/8-, >8-cell stage) at 46 and 60 h after IVF of unstained oocytes (US) and BCB-pos. oocytes “oocyte group +” and BCB-neg. oocytes “oocyte group –“, fertilised in vitro with oxidised sperm (OX S). Values are means + SD. Different letters “a, b” indicate differences (*p* < 0.05) between oocyte groups fertilised with OX S.

**Figure 4:**
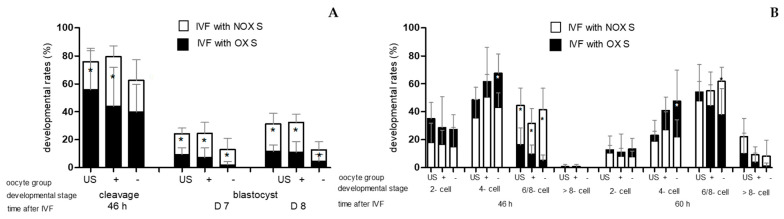
(**A**) Overlay of Figure 2A and Figure 3A with cleavage rates (46 h after IVF) and blastocyst rates on Days (D) 7 and 8 after IVF; (**B**) Overlay of Figure 2B and Figure 3B with percentages of embryos at different cell stages (2-, 4-, 6/8-, >8-cell stage) at 46 and 60 h after IVF of unstained oocytes (US) and BCB-pos. oocytes “oocyte group +” and BCB-neg. oocytes “oocyte group –“, fertilised in vitro with non-oxidised sperm (NOX S) (white bars) and oxidised sperm (OX S) (black bars). Values are means + SD; the asterisk “*” indicates differences (*p* < 0.05) between NOX S and OX S within oocyte groups.

After IVF with OX S, the cleavage rate did not differ (*p* ≥ 0.05) among the three oocyte group. If embryos were produced with OX S, blastocyst rates on D7 and D8 were lower (*p* < 0.05) in BCB-neg. oocytes compared to unstained oocytes and BCB-pos. oocytes. Between unstained oocytes and BCB-pos. oocytes, no differences in blastocyst rates on D7 and D8 (*p* ≥ 0.05) were observed after IVF with OX S (Figure 3A). Furthermore, when BCB-neg. oocytes were fertilised with OX S, at 60 h after IVF, more embryos were at the four-cell stage than unstained oocytes, which were fertilised with OX S (*p* < 0.05) (Figure 3B). In vitro fertilisation with OX S was followed by lower (*p* < 0.05) developmental rates (cleavage and blastocyst rates) than IVF with NOX S in all oocyte groups, with the exception of cleavage rate in BCB-neg. oocytes (*p* ≤ 0.05) (Figure 4A). BCB-stained oocytes (BCB-pos. oocytes/BCB-neg. oocyte) showed a significantly lower percentage of embryos cleaving ≤ 30 h after IVF than their counterparts produced with NOX S (Table 1). At 46 h after IVF, independent of oocyte class, fewer embryos were at the six- to eight-cell stage if produced with OX S instead of NOX S (*p* < 0.05). Additionally, in BCB-neg. oocytes fertilised with OX S, at 46 h and 60 h after IVF, more embryos were at the four-cell stage in comparison to embryos produced with BCB-neg. oocytes and NOX S (*p* < 0.05) (Figure 4B).

In order to investigate effects mediated by sperm exposed to oxidative stress, the decrease in developmental rates upon fertilisation with OX S in relation to those after fertilisation with NOX S was calculated for each oocyte quality group. The decrease in blastocyst rate on D7 was higher (*p* < 0.05) in BCB-neg. oocytes (85.94% ± 14.74%) than in unstained oocytes (60.37% ± 19.57%), but no differences (*p* ≥ 0.05) could be observed between other groups and other time points after IVF (Table 2).

### 3.3. Embryo Quality after Fertilisation with NOX S and OX S

The total cell number of embryos produced with unstained oocytes, BCB-neg. oocytes, and BCB-pos. oocytes fertilised with NOX S was not different (*p* ≥ 0.05) among oocyte quality groups (Table 3). Also, the mean apoptotic index did not differ (*p* ≥ 0.05) among groups. However, numerically more embryos of the BCB-neg. oocyte group had an apoptotic index > 5% compared to embryos of the groups unstained oocytes and BCB-pos. oocytes, fertilised with NOX S. The total cell number was significantly lower in embryos from BCB-neg. oocytes fertilised with OX S than in embryos from BCB-neg. oocytes fertilised with NOX S. Embryos of different oocyte classes produced with OX S did not differ in total cell number and apoptotic index (*p* ≥ 0.05).

## 4. Discussion

### 4.1. Embryo Development after Fertilisation of Oocytes of Different Qualities with NOX S

In our setup, around half of the oocytes stained blue (BCB-pos.) and the other half remained colourless (BCB-neg.), similar to reports from other authors [24,31]. But, the proportion can be different depending on the oocyte retrieval method [32,33,34]. The BCB-neg. oocytes had lower developmental rates after fertilisation with NOX S than control and BCB-pos. oocytes. This is in agreement with a number of studies in cattle [24,32,35,36,37,38,39] sheep [40,41], pigs [42,43,44], mice [34], horses [45] and goats [46].

Brilliant Cresyl Blue staining is used as an indicator for G6PDH activity. The biochemistry of BCB metabolism is not yet fully understood. It is hypothesised that BCB plays a role as an electron acceptor. The G6PDH catalyses the reaction of glucose-6-phosphate to 6-phosphoglucono-δ-lactone by reducing NADP to NADPH. During this reaction, an electron flow is induced which decolourises BCB [24]. Therefore, decolourisation of the BCB stain is directly linked to G6PDH activity and NADPH content. In addition, NADPH is needed for the regeneration of glutathione (an important antioxidant). Furthermore, activity of this enzyme is required for pentose and ribose-5-phosphate synthesis, and the last one is a precursor for the synthesis of nucleotides. A high enzyme activity in BCB-neg. oocytes has been described in growing and possibly also in degenerating oocytes [24,33,47] and was correlated to markers of low cytoplasmic maturity, as low levels of mitochondrial DNA replication factors [48], decreased cortical granule migration, peripheral mitochondria distribution [32], and low GSH content [31,49]. Furthermore, it was demonstrated that BCB-neg. oocytes are smaller and have the lowest penetrability for sperm [50]. What has not yet been studied is how these characteristics are biochemically related to G6PDH activity in oocytes and why, before maturation, a low enzyme activity is advantageous and a high enzyme activity is detrimental. This seems especially interesting as, during oocyte maturation, a high glucose-6-phosphate dehydrogenase activity is inevitable [51,52,53,54,55]. A possible explanation could be extrapolated from an established benefit of “quiet” metabolism in embryos [56]. There is recent evidence that this could be also true for oocytes [57].

Many authors observed a higher developmental competence of BCB-pos. oocytes compared with unstained oocytes [24,31,32,34,35,39,41,46]. However, in the present work and other studies [36,37,38,44,48] no significant differences between unstained oocytes and BCB-pos. oocytes were noticed. After fertilisation with one semen donor, BCB-pos. oocytes were yielding higher cleavage and blastocyst rates compared to fertilisation with another donor [37]. Studies showing no difference between BCB-pos. oocytes and unstained oocytes question the usefulness of the BCB test for IVP procedures and suggest that the hypothesis about enhancement and maximisation of overall embryo production efficiency by using the BCB staining in oocytes is not true [32,58,59]. Even negative effects of the BCB staining are reported [43,44,58]. In goat oocytes, signs of degeneration were observed after BCB staining in 3.6% of morphological superior oocytes. The authors hypothesised that some oocytes might be more prone to degeneration after BCB staining [58]. Also, exposure of oocytes to BCB after maturation resulted in impaired embryo development, suggesting that the toxic effect of BCB may depend on the stage of maturation of oocytes [44]. In a further study, BCB-exposed oocytes showed more chromosomal abnormalities than unstained oocytes. A higher susceptibility to the failure of first polar body extrusion, possibly related to an additional incubation time of 90 min necessary for BCB staining, was thought to be the cause for observed differences [43]. From the results of our study, a possible negative effect of staining can also be assumed, as developmental rates were numerically lower and the numbers of early-cleaving embryos were reduced after fertilisation with stressed sperm in BCB-pos. oocytes compared to unstained oocytes.

Our results confirm that the selection of oocytes according to the BCB staining does not improve the overall IVP outcome but allows an exclusion of oocytes with low developmental competence. How this lower developmental competence becomes manifest has been, to date, mainly studied on overall IVP outcome in form of blastocyst rate [59]. We also investigated the kinetics of embryo development. Interestingly, BCB-stained oocytes cleaved earlier than control oocytes. In other studies, BCB has been described as a stimulator of the pentose phosphate pathway [60,61]. A stimulation of oocyte metabolism could speed up embryo development. To the best of our knowledge, there are no other publications regarding possible stimulatory effects of the staining and, hence, the relevance of our observation is not clear to date. We also investigated the timing of embryo development at 46 and 60 h after IVF and found no difference between embryos produced from different oocyte quality classes. This is an interesting finding, since Opielia (2010) observed a lag of one cell cycle with a delay in reaching the expanded blastocyst stage in embryos derived from BCB-neg. oocytes [62]. In addition to the timing of embryo development, we also determined the quality of embryos produced with oocytes in relation to BCB staining. The total cell number did not significantly differ in blastocysts produced with unstained oocytes, BCB-pos. oocytes and BCB-neg. oocytes, as similarly reported in other studies [39]. Blastocysts’ total cell number is a marker for embryo quality, since it is believed that embryos with a higher cell number are more likely to implant and give rise to viable offspring [63]. In contrast to these findings, there are also studies which observed lower total cell numbers in BCB-neg. oocytes [24,35,36]. The mean apoptotic indices in blastocysts produced with BCB-pos. oocytes, BCB-neg. oocytes and unstained oocytes were similar in our study. Cell death is a normal feature of embryonic development and allows elimination of damaged cells. Embryos in developmental arrest show high rates of apoptotic cells (reviewed in [64]). However, the percentage of blastocysts with a high apoptotic index was higher in BCB-neg. oocytes than in unstained oocytes and BCB-pos. oocytes, suggesting a trend towards an increased rate of apoptosis in embryos produced from BCB-neg. oocytes. Increased rates of apoptosis in BCB-neg. oocytes were also observed in other studies applying the TUNEL assay in bovine embryos produced with BCB-selected oocytes [36].

### 4.2. Embryo Development after Fertilisation of Oocytes of Different Qualities with OX S

As BCB staining is selective for G6PDH activity, we hypothesised that there is an association between BCB staining of the oocytes and their competence for embryo development after fertilisation with oxidatively damaged sperm, because of the enzyme’s role in providing the oocyte with antioxidants.

We observed that, if oocytes (independent of quality class) were fertilised with OX S, developmental rates on D7 and D8 were significantly reduced in comparison to fertilisation with NOX S. The BCB-neg. oocytes showed the highest decrease in D7 blastocyst rates, with rates close to zero upon fertilisation with OX S, and showed a delayed embryo development at 46 and 60 h after IVF. Furthermore, the cell number in blastocysts produced with BCB-neg. oocytes and OX S was lower than in the embryos produced with NOX S and BCB-neg. oocytes.

The observation that developmental rates are decreased and delayed after fertilisation with oxidised semen are consistent with previous studies [21,65,66,67,68]. In BCB-neg. oocytes, the developmental decrease was the most severe. A possible reason for a decreased competence of BCB-neg. oocytes, in regard to supporting embryo development with damaged sperm, could be an immaturity of these oocytes, which involves possibly inferior equipment with DNA damage repair proteins. This has been already hypothesised by Rahman et al. (2012), who observed a better IVP outcome if large, instead of small, oocytes were fertilised with hydrogen-peroxide-treated sperm. In consequence, it was proposed that damaged sperm cannot be repaired by small oocytes and embryo development fails [21]. Another explanation for the lower competence to support embryo development could be an alteration in the oocytes’ redox state. Stimulation of the pentose phosphate pathway in maturing bovine oocytes induced a shift in the redox state towards a more oxidised state. It was proposed that oxidation of developmentally important molecules may contribute to subsequently impaired embryo development [53]. This goes in line with the finding of reduced GSH levels in BCB-neg. oocytes [31]. This GSH detoxifies hydrogen peroxide and lipid peroxides via the action of the glutathione peroxidase. Oxidatively damaged sperm probably also introduce toxic metabolites upon fertilisation. Oocytes equipped with low antioxidant capacities might be not able to detoxify these and become damaged, with consequences for further embryo development. How a high G6PDH activity and low GSH levels are linked in oocytes has not been discussed so far. In HeLa cells, it has been shown that low levels of GSH induce an increase in G6PDH expression, in order to restore GSH levels [69]. Furthermore, it can be hypothesised that oocytes with a high enzyme activity are already suffering from oxidative stress and used up its GSH pool. As a consequence, an introduction of oxidised sperm might overwhelm the anti-oxidative defence system, finally leading to developmental arrest. Interestingly, oocytes with a low enzyme activity (BCB-pos. oocytes) were not from superior quality, in comparison to unstained oocytes, in regard to supporting embryo development after fertilisation with sperm exposed to oxidative stress in any of the parameters, as it might be expected if high G6PDH activity is detrimental. A possible explanation could be that BCB staining itself negatively influences embryo development, as discussed above.

## 5. Conclusions

A high G6PDH activity assessed by BCB staining in oocytes is related to a low competence to support embryo development after fertilisation with oxidised sperm. This possibly implies that BCB-neg. oocytes are less equipped with factors which repair damaged sperm, and, hence, fail to support embryo development. Future studies should evaluate which factors mediate the lower competence to support embryo development after fertilisation with oxidised sperm. Also, in the view of increased fertility problems in human medicine [70,71], understanding the oocytes’ competence to deal with damaged sperm would potentially allow to develop techniques to increase the oocytes’ repair capacity in vivo or in vitro [25]. Furthermore, the exact mechanisms of low and high G6PDH activity and correlation to BCB stainability in oocytes should be clarified, especially considering the inconsistency in reports to the quality of BCB-pos. oocytes.

## Figures and Tables

**Table 1:** Early-cleaving embryos (=percentage of embryos cleaved at 30 h/percentage of embryos cleaved at 46 h) originating from unstained oocytes (US) and stained oocytes (BCB-pos., BCB-neg.) fertilised with non-oxidised sperm (NOX S) and oxidised sperm (OX S). Values are means ± SD. The asterisk “*” indicates differences (*p* < 0.05) between NOX S and OX S within oocyte groups. Different lowercase letters “^a^, ^b^, ^c^” indicate differences (*p* < 0.05) between oocyte groups fertilised with NOX S. Different capital letters “^A^, ^B^” indicate differences (*p* < 0.05) between oocyte groups fertilised with OX S.

		Early-Cleaving Embryos	
	Sperm	NOX S (N = 228)	OX S (N = 263)
Oocyte			
US (N = 181)		46.91 ± 16.83 ^a^	41.23 ± 12.57 ^A^
BCB-pos. (N = 154)		75.93 ± 11.27 ^b^*	12.94 ± 13.74 ^B^*
BCB-neg. (N = 248)		62.46 ± 14.45 ^bc^*	12.40 ± 5.44 ^B^*

**Table 2:** Decrease in developmental rates (cleavage rates (CR) and blastocyst rates (BR)) upon fertilisation of different oocyte classes (unstained oocytes (US) and BCB-pos. oocytes “oocyte group +” and BCB-neg. oocytes “oocyte group −”) fertilised with oxidised sperm (OX S), in comparison to fertilisation with non-oxidised sperm (NOX S). Formula: 100-(developmental rate [cleavage rate, blastocyst rate] with OX S/developmental rate [cleavage rate, blastocyst rate] with NOX S). Values are means ± SD. Different letters “^a^, ^b^” indicate differences (*p* < 0.05) between oocyte groups.

Oocyte	Decrease in CR (%)	Decrease in BR D7 (%)	Decrease in BR D8 (%)
US	27.63 ± 23.70 ^a^	60.37 ± 19.57 ^a^	62.84 ± 13.89 ^a^
BCB-pos.	46.10 ± 32.70 ^a^	67.05 ± 39.33 ^ab^	66.56 ± 25.65 ^a^
BCB-neg.	37.83 ± 27.61 ^a^	85.94 ± 14.74 ^b^	77.04 ± 22.45 ^a^

**Table 3:** Total cell number, apoptotic index and percentage of blastocysts with an apoptotic index > 5% (%) in blastocysts on D7, after fertilisation of unstained oocytes (US) and stained oocytes (BCB-pos., BCB-neg.) with non-oxidised sperm (NOX S) and oxidised sperm (OX S). Values are means ± SD. The asterisk “*” indicates differences (*p* < 0.05) between NOX S and OX S within oocyte groups.

	Embryos (n)		Total Cell Number		Apoptotic Index (%)		Percentage of Embryos with an Apoptotic Index > 5% (%)	
Oocyte	NOX S	OX S	NOX S	OX S	NOX S	OX S	NOX S	OX S
US	16	23	153.90 ± 47.20	140.50 ± 28.47	4.70 ± 3.69	4.47 ± 3.90	38	34
BCB-pos.	17	10	146.10 ± 43.30	147.30 ± 38.65	6.73 ± 7.48	6.25 ± 12.63	44	30
BCB-neg.	14	13	145.90 ± 24.62 *	124.90 ± 28.13 *	10.10 ± 11.29	5.82 ± 9.41	64	23

## Data Availability

All data are presented here.
